# Thoracic Curve Correction Ratio: An Objective Measure to Guide against Overcorrection of a Main Thoracic Curve in the Setting of a Structural Proximal Thoracic Curve

**DOI:** 10.3390/jcm11061545

**Published:** 2022-03-11

**Authors:** Matthew R. Landrum, Andrew H. Milby, Burt Yaszay, Stefan Parent, Susan E. Nelson, Joshua M. Pahys, Amer F. Samdani, Anthony C. Capraro, John M. Flynn, Patrick J. Cahill, on behalf of the Harms Study Group and Harms Non-Fusion Study Group

**Affiliations:** 1 Division of Orthopaedics, Children’s Hospital of Philadelphia, Philadelphia, PA 19104, USA; landrumm2@uthscsa.edu (M.R.L.); susan_nelson@urmc.rochester.edu (S.E.N.); flynnj@email.chop.edu (J.M.F.); 2 Department of Orthopaedics, University of Texas Health Sciences Center, San Antonio, TX 78229, USA; 3 Department of Orthopaedics, Emory University School of Medicine, Atlanta, GA 30322, USA; andrewmilby@gmail.com; 4 Department of Orthopedics and Sports Medicine, Seattle Children’s Hospital, Seattle, WA 98105, USA; byaszay.rady@gmail.com; 5 Orthopedics-CHU Sainte-Justine, Montreal, QC H3T 1C5, Canada; stefan.parent@umontreal.ca; 6 Department of Orthopaedics, University of Rochester Medical Center, University of Rochester, Rochester, NY 14642, USA; 7 Shriners Hospitals for Children, Philadelphia, PA 19140, USA; jpahys@shrinenet.org (J.M.P.); asamdani@shrinenet.org (A.F.S.); 8 School of Osteopathic Medicine, Rowan University, Glassboro, NJ 08028, USA; acapraro24@gmail.com; 9 Department of Orthopaedic Surgery, University of Pennsylvania, Philadelphia, PA 19104, USA

**Keywords:** thoracic curve correction ratio (TCCR), T1 tilt, idiopathic scoliosis, posterior spinal fusion (PSF), Lenke 2 curve correction

## Abstract

Purpose: The correction of double thoracic (Lenke 2) curves has been associated with higher rates of postoperative shoulder imbalance that may compromise long-term outcomes following spinal deformity correction. A number of methods have been proposed to mitigate this risk, though no accepted standard measurement exists. The purpose of this study is to validate a novel quantitative method of determining the relative curve correction magnitude in double thoracic curves. Methods: Retrospective data from a multi-center database of patients undergoing surgical correction of left-proximal thoracic, right-main thoracic Lenke 2 curves were analyzed. A novel measurement tool, the Thoracic Curve Correction Ratio (TCCR), was applied for the purposes of validation against historical data. Results: A total of 305 patients with complete two-year follow-up data were included. The TCCR, or the ratio of postoperative percent correction of the thoracic curves divided by the ratio of the preoperative curve magnitudes, displayed a significant negative correlation (Pearson R = −0.66; *p* < 0.001) with T1 tilt at two years postoperatively. Conclusions: The TCCR could be added as an important factor in the preoperative planning process and intraoperative assessment in order to reduce postoperative T1 tilt. While T1 tilt remains an imperfect surrogate measure for clinical shoulder balance, it serves as one of many potential measures that the surgeon may evaluate quantitatively and radiographically.

## 1. Introduction

Modern spinal instrumentation and fusion techniques have allowed for the application of increasingly powerful correction maneuvers during spinal deformity surgery. As these techniques have evolved, so too has the planning process for such procedures. The clinical significance of the proximal thoracic curve with regard to shoulder balance was first highlighted in the literature by Ponseti [1] and has been subsequently recognized as an important consideration in the prevention of postoperative shoulder imbalance following surgical correction [2,3,4]. Persistent shoulder imbalance following posterior spinal fusion (PSF) for deformity correction may have a significant negative impact on clinical outcomes and has led to a number of proposed measures for quantifying its presence and impact. Kuklo et al. sought to improve upon the historically used T1 tilt by studying the correlation of numerous radiographic surrogates with clinical shoulder balance and concluded that the clavicle angle was the most useful [5]. Yagi et al. proposed a variation on this technique, instead examining the clavicle chest cage angle difference (CCAD) [6]. The authors noted that, in their series, the patients in the unbalanced group based upon CCAD measurements were more likely to report clinical dissatisfaction with their appearance. Smyrnis et al. proposed the First Rib Index (FRI) as another radiographic surrogate measure for shoulder balance [7]. Importantly, they noted that satisfaction with shoulder balance was dependent on the body habitus, with thinner patients being less tolerant of radiographic or clinically-apparent imbalance. In contrast to Kuklo et al., the authors also noted the utility of intraoperative T1 tilt and the correlation of reduced T1 tilt and achievement of clinical shoulder balance in their series.

Double thoracic (Lenke 2) curves represent one of the most challenging curve types in which to obtain satisfactory correction of shoulder balance [8]. Clinical shoulder balance remains difficult to assess and quantify; residual T1 tilt may increase the risk of postoperative shoulder imbalance, though this relationship has not been consistently observed in the literature [9,10,11]. Despite the utilization of modern segmental instrumentation, Chang et al. reported on the relative ineffectiveness of vertebral derotation maneuvers on the proximal thoracic curve, further highlighting the importance of strategically avoiding overcorrection of the main thoracic curve so as to minimize the risk of worsening shoulder imbalance [12]. Accepting that no perfect radiographic surrogate measure for clinical shoulder balance exists, we instead sought to determine whether a consistent and reliable measure, such as T1 tilt, could be used to quantitatively assess the effect of relative proximal and main thoracic curve correction. Utilizing T1 tilt as a radiographic surrogate for shoulder height, we hypothesized that a mismatch between preoperative T1 tilt and the ratio of proximal thoracic to main thoracic curve correction may be associated with residual T1 tilt following spinal deformity correction.

## 2. Materials and Methods

We performed a retrospective review of data from a prospective, multi-center AIS database. All PSF performed from July 1996 to May 2013 for left proximal-thoracic, right main-thoracic, left lumbar Lenke 2 idiopathic curves with a minimum of two-year follow-up were analyzed. Curves were considered structural according to the Lenke classification criteria [8]. These restrictive inclusion criteria were used to facilitate quantitative analysis. Primary measures included Proximal Thoracic (PT) and Main Thoracic (MT) Cobb angles, as well as T1 tilt (left shoulder up = +, by convention), from standing radiographs at the preoperative and two-year postoperative time points. SRS-24 self-image, function, and satisfaction scores were obtained during follow-up in an attempt to correlate clinical outcome scores.

**Figure 1:**
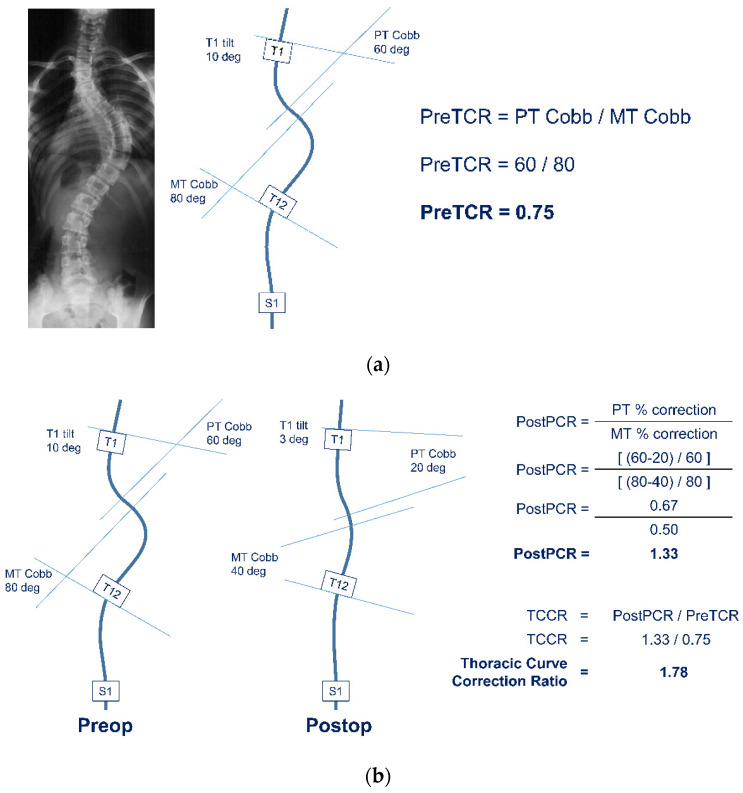
(**a**) Case example demonstrating calculation of Preoperative Thoracic Curve Ratio (PreTCR) from the Proximal Thoracic (PT) and Main Thoracic (MT) Cobb angles. (**b**) Continuation of case example from Figure 2a demonstrating calculation of postoperative Thoracic Curve Correction Ratio (TCCR).

From these data, the following additional three parameters were calculated:

(1) Preoperative Thoracic Curve Ratio (PreTCR) = [PT Cobb/MT Cobb] (Figure 1a)
(2) Postoperative Percent Correction Ratio (PostPCR) = [(PT % Correction)/(MT % Correction)] (Figure 1b)
(3) Thoracic Curve Correction Ratio (TCCR) = [PostPCR/PreTCR] (Figure 1b)

Descriptive statistics were generated for baseline demographic variables. All statistical analyses were performed in PASW Statistics, version 18. *p*-values less than 0.05 were considered significant.

## 3. Results

**Figure 2:**
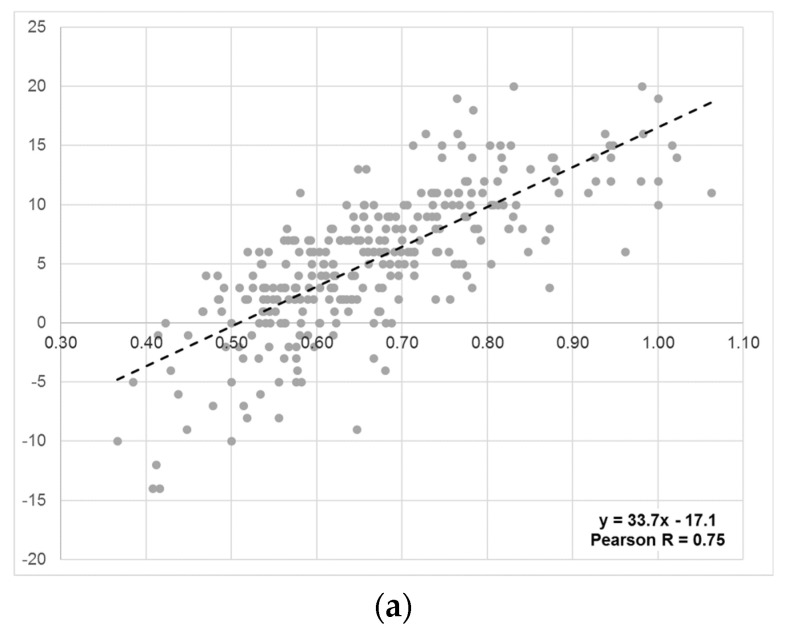

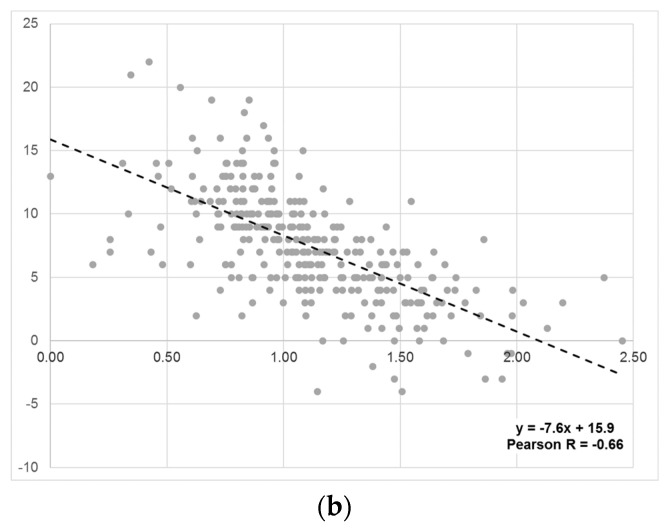
(**a**) Preoperative Thoracic Curve Ratio (PreTCR, *X* axis) vs. Preoperative T1 Tilt Angle (*Y* axis). (**b**) Thoracic Curve Correction Ratio (Percent Correction Ratio/PreTCR, *X* axis) vs. Postoperative T1 Tilt Angle (*Y* axis).

A total of 305 patients with complete two-year follow-up data were included in the analysis. Demographic characteristics of the overall cohort are reported in Table 1. The ratio of the proximal thoracic Cobb angle to the main thoracic Cobb angle (PreTCR) displayed a positive correlation (Pearson R = 0.75; *p* < 0.001) with T1 tilt (Figure 2a). The ratio of postoperative percent correction of these curves (PostPCR) divided by the PreTCR displayed a negative correlation (Pearson R = −0.66; *p* < 0.001) with T1 tilt at two years postoperatively (Figure 2b). No significant associations were observed between the TCCR and clinical outcome scores, including SRS-24 satisfaction, and the changes in SRS-24 self-image and function domains over the follow-up period (Table 2).

## 4. Discussion

Complex spinal deformity correction remains both an art and a science. There exists a paucity of reliable, reproducible measures that can be used for quantitative preoperative planning and intraoperative assessment. While not applicable in every case due to patient-specific anatomic variation, the T1 tilt remains a useful measure given its reliability and ease of measurement with standard intraoperative imaging techniques. Its combination with the widely-used Cobb angle assessment of coronal plane deformity offers an appealing combination of factors to aid the surgeon in planning a correction strategy.

The PreTCR measurement demonstrated a significant positive correlation with T1 tilt in our series. This correlation suggests that this measure may be of use in determining whether a proximal thoracic curve is structural and merits inclusion in the correction and fusion construct. The selection of surgical levels was not directly assessed in this study and may have a large potential impact on the extent and durability of postoperative correction. This remains another potential application of such quantitative measures that merits further analysis.

Ultimately the TCCR is intended as a tool to aid in the determination of the relative extent of PT and MT coronal plane correction in patients’ double thoracic (Lenke 2) curves. While prior studies have shown that over-correction of the main thoracic curve compared to the proximal thoracic curve can lead to shoulder imbalance, it is the author’s goal to quantify this mismatch [13,14]. The TCCR measure displayed a significant negative correlation with postoperative T1 tilt at final follow-up. While the TCCR is a post-hoc measure, knowledge of its negative correlation with postoperative T1 tilt may enable the surgeon to modify the preoperative correction strategy based upon a case-specific idealized ratio of PT and MT percent correction. Application of the TCCR suggests that patients with a significant proximal thoracic curve should undergo asymmetric over-correction of the PT curve relative to the MT curve that is greater than the ratio of the preoperative curve magnitudes in order to reduce the likelihood of residual postoperative T1 tilt. This may be of benefit in the reduction of clinically significant postoperative shoulder imbalance, though the surgeon is cautioned to determine whether or not an individual patient’s preoperative T1 tilt correlates with their clinical assessment of shoulder balance before aiming solely to minimize this single radiographic value. This is similar to the planning process for correction of Lenke 3 curves, wherein overcorrection of the lumbar curve relative to the stiffer thoracic curve may also lead to an inadvertent worsening of coronal balance.

**Figure 3:**
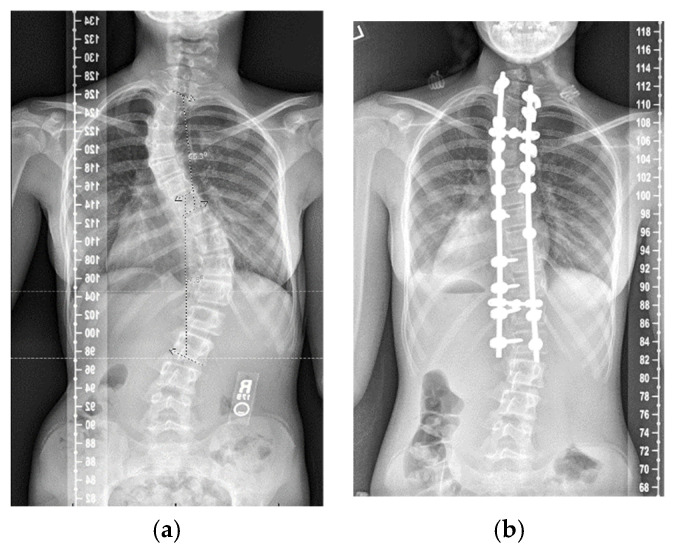
(**a**) Patient with PT curve of 55° and MT curve of 57°. Their PreTCR is 0.96. (**b**) T2-L1 posterior spinal fusion with PostPCR 0.64 and TCCR 0.66. There is continued T1 tilt and the left shoulder is high.

For example, the patient in Figure 3a had a PT curve of 55° and an MT curve of 57°, making their PreTCR 0.96. Their preoperative T1 tilt was 15° and their left shoulder was high. They underwent posterior instrumented fusion from T2 to L1. Their post-correction PT curve (Figure 3b) was 29° and the MT curve was 15°. Their percent correction of the PT curve was 47.3% and the MT curve was 73.7%. Their PostPCR was 0.64 and their TCCR was 0.66. Postoperatively, their left shoulder was still high and the T1 tilt was 11°. When utilizing the TCCR, the operative surgeon could note that a more aggressive correction of the PT curve relative to the MT curve would potentially improve T1 tilt, thereby leveling the shoulders. In the setting of an extremely stiff proximal thoracic curve, the operating surgeon could decrease coronal correction of the main thoracic curve to avoid driving the shoulder higher.

This study is a preliminary validation of the TCCR concept in a highly-standardized group of left PT, right MT, double thoracic idiopathic scoliosis cases, and as such is not without significant limitations. These strict selection criteria, utilized in order to simplify the quantitative analysis, limit the generalizability of this technique beyond cases of this nature. Most importantly, the concept of shoulder balance following spinal deformity correction remains controversial and elusive to standardized clinical or radiographic measures. While a number of measures have since superseded T1 tilt in the literature, there exists no consensus on the ideal set of parameters to ensure patient satisfaction from a cosmetic and functional perspective [9,15,16,17]. As such, the use of the TCCR remains at the surgeon’s discretion should postoperative achievement of neutral T1 tilt be deemed beneficial based upon preoperative assessment of the individual patient’s thoracic and shoulder girdle morphology.

In patients with double thoracic curves, the correction of the PT curve relative to the MT curve may be modified based on the ratio of the preoperative curve magnitudes in order to reduce the likelihood of residual postoperative T1 tilt. This may, in turn, aid in the achievement of postoperative shoulder balance in select cases.

## Figures and Tables

**Table 1:** Demographic characteristics.

Variable	*n*	(%)			
Gender					
Male	98	(32)			
Female	207	(68)			
Curve Type					
Lenke 2A	213	(70)			
Lenke 2B	57	(19)			
Lenke 2C	35	(11)			
	*n*	Mean	±	SD	(Range)
Age (years)	305	14.5	±	2.2	(10.1–21.5)
Body Mass Index	260	22.0	±	4.1	(13.1–41.8)
Preop PT Cobb	305	38.9	±	8.6	(25.0–86.0)
Preop MT Cobb	305	59.1	±	11.5	(36.0–108.0)
Follow-up (years)	305	2.3	±	0.4	(1.9–4.4)
Final PT Cobb	305	21.0	±	7.4	(2.0–55.0)
% PT Correction	305	46	±	16	(0–94)
Final MT Cobb	305	21.2	±	7.8	(3.0–49.0)
% MT Correction	305	64	±	13	(12–95)

SD: standard deviation, PT: proximal thoracic, MT: main thoracic.

**Table 2:** Correlations with Clinical Outcome Scores.

Variable	*n*	Correlation with TCCR	
		Pearson R	*p* value
Change in SRS-24 Self Image	234	0.11	0.08
Change in SRS-24 Function	233	0.08	0.20
SRS-24 Satisfaction	234	0.07	0.25

TCCR: thoracic curve correction ratio, SRS: Scoliosis Research Society.

## Data Availability

Restrictions apply to the availability of the data. Data was obtained from the Harms Study Group AIS Database and are available with the permission of the Harms Study Group.

## Supplementary Materials

The following supporting information can be downloaded at: https://www.mdpi.com/article/10.3390/jcm11061545/s1.
