# Supplementary material for: Thoracic Curve Correction Ratio: An Objective Measure to Guide against Overcorrection of a Main Thoracic Curve in the Setting of a Structural Proximal Thoracic Curve
Source: J Clin Med. 2022 Mar 11;11(6):1545. doi: 10.3390/jcm11061545 (PMC8954637; doi:10.3390/jcm11061545)
Supplement: Supplementary file 1 [file jcm-11-01545-s001.zip › jcm-1515551-supplementary.pdf]

Harms Study Group Investigators: Aaron Buckland, MD; New York University; Amer Samdani, MD; Shriners Hospitals for Children—Philadelphia; Amit Jain, MD; Johns Hopkins Hospital; Baron Lonner, MD; Mount Sinai Hospital; Benjamin Roye, MD; Columbia University; Burt Yaszay, MD; Rady Children's Hospital; Chris Reilly, MD; BC Children's Hospital; Daniel Hedequist, MD; Boston Children's Hospital; Daniel Sucato, MD; Texas Scottish Rite Hospital; David Clements, MD; Cooper Bone & Joint Institute New Jersey; Firoz Miyanji, MD; BC Children's Hospital; Harry Shufflebarger, MD; Paley Orthopedic & Spine Institute; Jack Flynn, MD; Children's Hospital of Philadelphia; John Asghar, MD; Paley Orthopedic & Spine Institute; Jean Marc Mac Thiong, MD; CHU Sainte-Justine; Joshua Pahys, MD; Shriners Hospitals for Children—Philadelphia; Juergen Harms, MD; Klinikum Karlsbad-Langensteinbach, Karlsbad; Keith Bachmann, MD; University of Virginia; Lawrence Lenke, MD; Columbia University; Lori Karol, MD; Children's Hospital, Denver Colorado; Mark Abel, MD; University of Virginia; Mark Erickson, MD; Children's Hospital, Denver Colorado; Michael Glotzbecker, MD; Rainbow Children's Hospital, Cleveland; Michael Kelly, MD; Washington University; Michael Vitale, MD; Columbia University; Michelle Marks, PT, MA; Setting Scoliosis Straight Foundation; Munish Gupta, MD; Washington University; Nicholas Fletcher, MD; Emory University; Noelle Larson, MD; Mayo Clinic Rochester Minnesota; Patrick Cahill, MD; Children's Hospital of Philadelphia; Paul Sponseller, MD; Johns Hopkins Hospital; Peter Gabos, MD: Nemours/Alfred I. duPont Hospital for Children; Peter Newton, MD; Rady Children's Hospital; Peter Sturm, MD; Cincinnati Children's Hospital; Randal Betz, MD; Institute for Spine & Scoliosis; Stefan Parent, MD: CHU Sainte-Justine; Stephen George, MD; Nicklaus Children's Hospital; Steven Hwang, MD; Shriners Hospitals for Children—Philadelphia; Suken Shah, MD; Nemours/Alfred I. duPont Hospital for Children; Sumeet Garg, MD; Children's Hospital, Denver Colorado; Tom Errico, MD; Nicklaus Children's Hospital; Vidyadhar Upasani, MD; Rady Children's Hospital

Harms Non-Fusion Study Group Investigators: Amer Samdani, MD; Shriners Hospitals for Children—Philadelphia; Ahmet Alanay, MD; Acibadem Maslak Hospital, Turkey; Baron Lonner, MD; Mount Sinai Hospital; Bob Cho, MD; Shriner's Pasadena CA; Burt Yaszay, MD; Rady Children's Hospital; Caglar Yilgor, MD; Acibadem Maslak Hospital, Turkey; Dan Hoernschmeyer, MD; University of Missouri Health Care; Firoz Miyanji, MD; BC Children's Hospital; Harry Shufflebarger, MD; Paley Orthopedic & Spine Institute; John Asghar, MD; Paley Orthopedic & Spine Institute; Josh Murphy, MD; Children's Healthcare of Atlanta; Kevin Neal, MD; Nemours Children's Clinic, Jacksonville; Laurel Blakemore, MD; Pediatric Specialists of Virginia/Children's National; Lawrence Haber, MD; Ochsner health center for children New Orleans; Noelle Larson, MD; Mayo Clinic Rochester Minnesota; Patrick Cahill, MD; Children's Hospital of Philadelphia; Peter Newton, MD; Rady Children's Hospital; Stefan Parent, MD: CHU Sainte-Justine; Suken Shah, MD; Nemours/Alfred I. duPont Hospital for Children
